# Polystyrene surface modification using excimer laser and radio-frequency plasma: blood compatibility evaluations

**DOI:** 10.1186/2194-0517-1-4

**Published:** 2012-11-14

**Authors:** Shadab Bagheri-khoulenjani, Hamid Mirzadeh

**Affiliations:** Department of Polymer Engineering, Amirkabir University of Technology, 424 Hafez-Ave., P.O. Box 15875-4413, Tehran, Iran

**Keywords:** Polystyrene, RF plasma, Excimer laser, Hydrophobicity, Hydrophilicity, Morphology, Blood compatibility

## Abstract

Biomaterial surface modification is an efficient method to improve and control blood component-material interactions. In the present study, two different methods (ArF excimer laser irradiation and radio-frequency (RF) plasma treatment) were applied in separate procedures to create a vast range of physicochemical characteristics on the surface of polystyrene (PS) and investigate their effects on blood compatibility of treated surfaces. Atomic force microscopy (AFM) and Fourier transmission infrared analysis were applied to study the morphology and chemical characteristics of treated samples in comparison with those of the untreated PS. Contact angle and surface tension measurements with two different solvents were used to evaluate the wettability and surface energy of the treated PS films. The effect of the physicochemical properties of the PS surface on blood compatibility was investigated using lactate dehydrogenase (LDH) method. AFM studies showed that after laser treatment, some distinctive nanostructures are created on the surface of PS. The data from contact angle measurements demonstrated that ArF excimer laser irradiation and RF plasma treatment created surfaces with a vast range of properties in the wettability point of view. The LDH results revealed that after surface modification by laser irradiation and plasma treatment, blood compatibility of PS films was enhanced. In addition, these results offered that the most blood compatible samples were those which irradiated with 5 pulses of laser and the one treated 4 minutes in oxygen plasma.

**Electronic supplementary material:**

The online version of this article (doi:10.1186/2194-0517-1-4) contains supplementary material, which is available to authorized users.

## Background

Although synthetic polymers are widely used in medicine, in direct contact with blood, these polymers are still prone to initiate the formation of clots due to activation of platelets and other components of the blood coagulation system ([Aiping and Tian 2006]). It is well known that interactions between the surface of an artificial biomaterial and biological environment are the key factor to determine the biocompatibility (Mirzadeh and Dadsetan 2003Khorasani and Mirzadeh 2004aChu 2007Mirzadeh and Bagheri 2007Chen et al. 2008) and blood compatibility (Khorasani and Mirzadeh2004bAiping and Tian 2006Chen et al.^2008^) of materials. Various studies have shown that the interfacial interactions are related to surface properties, such as surface chemical composition (Jung et al. 2009), surface wettability (Ikada et al. 1981,1983), surface morphology (Dadsetan et al. 2001a), and surface charge (Corum and Hlady 2010).

Ikada et al. 1981,1983) have shown that wettability can affect the blood compatibility throughout the adsorption of the protein on the polymer surface. They showed that in both superhydrophilic and superhydrophobic surfaces, protein adsorption is in the minimum level. Meanwhile, it has been shown that surface morphology can affect the blood compatibility of the polymers. Surface morphologies in the nanoscales are more blood compatible than those in the microscales due to less entrapment of platelets in the surface mounds (Dadsetan et al.2001b).

To improve the blood compatibility of biomaterials, various techniques of surface modification have been applied in order to alter the surface properties without affecting the bulk ones (Dadsetan et al.2001aKarkhaneh et al. 2010Solouk et al.2011a). Among these techniques which have been developed for this purpose, laser irradiation (Mirzadeh et al.^1993^,^2011^Khorasani and Mirzadeh [Bibr CR13][Bibr CR16]) and plasma treatment (Chu 2007Mochizuki et al.^2011^Solouk et al.[Bibr CR24][Bibr CR25]) can be applied to create new functional groups, micro- and nanostructures, and change in surface wettability.

The purpose of this study was to provide the PS surfaces with a wide range of wettability and morphological properties using ArF excimer laser irradiation and RF plasma treatment with different gases (oxygen and argon), followed by studies on their eventual physicochemical characteristics in relation to blood compatibility. To the best of our knowledge, no work has been reported in the literature dealing with comparison between laser and RF plasma effects on both hydrophobicity and hydrophilicity of PS surfaces in the blood compatibility point of view.

## Results and discussion

### Physicochemical properties

**Figure 1:**
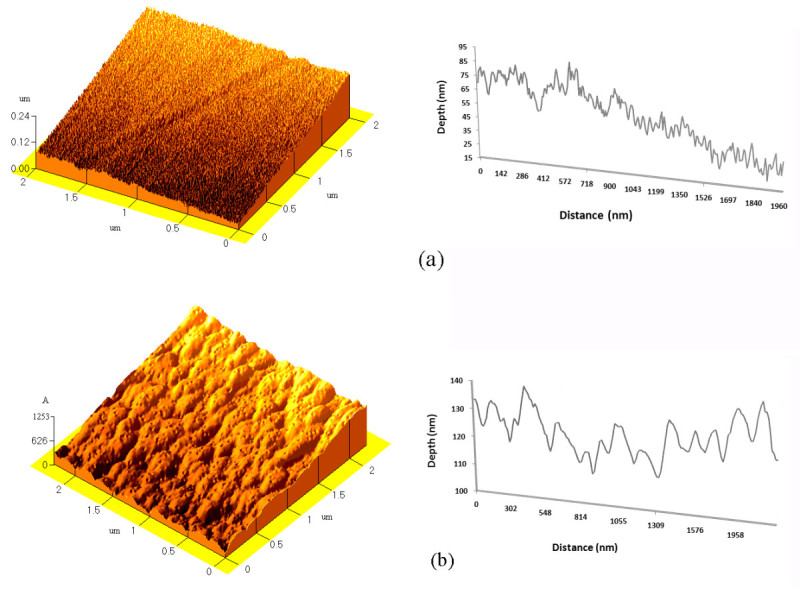
**AFM image and profile of (a) untreated PS and (b) one-pulse-laser-treated PS.**

The physicochemical characteristics of PS surface treated with laser and plasma are reported elsewhere thoroughly (Mirzadeh and Bagheri 2007). Briefly, the scanning electron microscopy (SEM; not reported here) and AFM studies (Figure 1) of laser-irradiated PS revealed that laser irradiation created some nanostructures (with average valley width of 150 nm and average depth of 15 nm) on the PS surface. It has been reported that the laser irradiation of polymers such as polystyrene with high adsorption results in ablation of the surface which subsequently leads to induction of some specific morphological structures on the surface (Srinivasan et al. 1990Knittel et al.^1997^Dadsetan et al.2001b). In addition, increasing pulse numbers causes augmentation of the valley size (Mirzadeh and Bagheri 2007).

**Figure 2:**
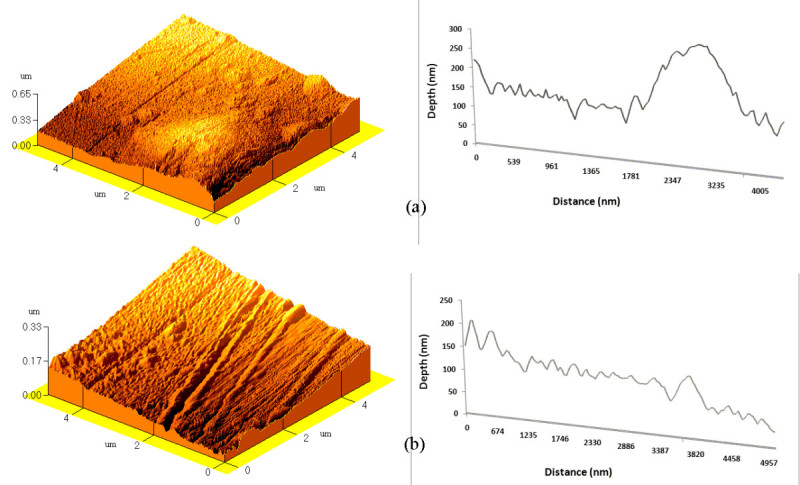
**AFM image and profile of (a) 15-min Ar plasma-treated and (b) 4-min O**_**2**_ **plasma-treated PS.**

Surface roughness of polystyrene increased after exposure to argon and oxygen plasma (Figure 2).

This can be related to two different phenomena: chemical etching and physical spattering of the polystyrene surface subjected to highly energetic ions and radicals of plasma. Exposure to the active residuals and ions of plasma could result in dissociation of some chemical bonds from the surface of the polymer, which results in ablation and changes in the morphology of the surface. In addition, collision of highly energetic specimens of plasma to the surface can cause some etching on the surface of polystyrene (Ingaki 1996).

SEM studies (reported elsewhere (Mirzadeh and Bagheri 2007)) showed that by increasing the time of exposure of samples to argon and oxygen plasma, roughness and morphological changes of the polystyrene surface were accentuated (Mirzadeh and Bagheri 2007). The SEM and AFM studies revealed that morphological changes of polystyrene after laser irradiation were more significant than those after argon and oxygen plasma treatments. It can be due to less sensitivity of polystyrene to plasma (Ingaki 1996) and high adsorption of UV radiations at 193 nm by polystyrene (Rabek 1995,1996).

**Figure 3:**
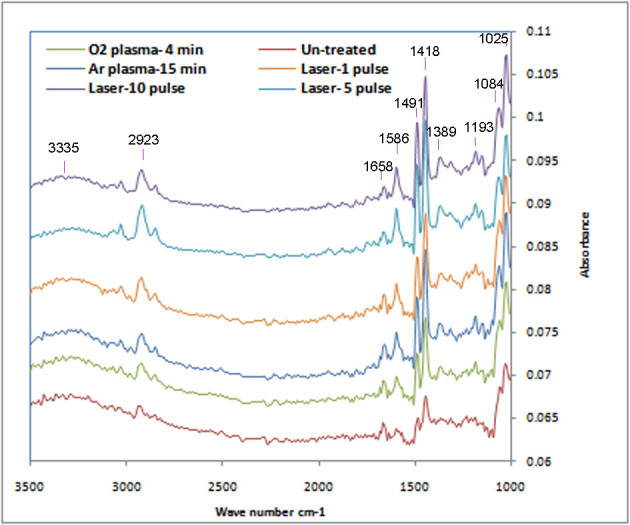
**ATR-FTIR spectra of untreated PS and PS treated with laser and plasma.**

The attenuated total reflectance-Fourier transform infrared (ATR-FTIR) studies showed that some oxygen-based functional groups were introduced into the PS surfaces after laser irradiation and plasma treatment (Figure 3). Peaks at 1,600 and 3,284 cm^−1^ related to -OH groups and those at 1,165 and 1,388 cm^−1^ attributed to -O-O- groups (Mirzadeh and Bagheri 2007) were observed in the spectra of all laser- and plasma-treated samples.

Rabek (1995,1996) showed that formation of oxygen-based functional groups on the polystyrene surface after laser irradiation is due to adsorption of photons by a benzene ring and excitation of its C-H bond which results in formation of radicals on the polymer chain. Interaction of oxygen existing in the air with these radicals induces formation of oxygen-based functional groups on the surface of polystyrene. Formation of these functional groups has been reported by Lu et al. (2004) and Zhu et al., too.

Exposure of polystyrene to O_2_ plasma led to formation of oxygen-based functional groups on the surface due to collision of the radicals or active components of oxygen to the polystyrene surface. Formation of oxygen-based functional groups onto the polystyrene surface after argon plasma modification could occur via post-oxidation reactions. During exposure to argon plasma, some stable radicals were created on the polystyrene surface. When exposed to the air, survived radicals reacted with O_2_ molecules of the air and oxygen-based functional groups were formed.

**Figure 4:**
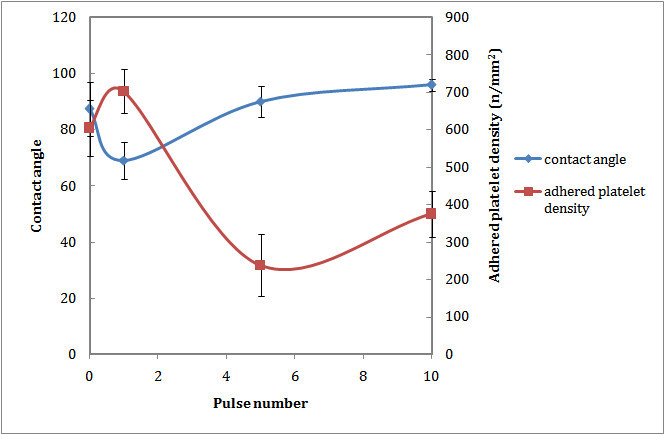
**Wettability and blood compatibility of PS after laser irradiation.**

Figure 4 shows that contact angle of water on the surface of the laser-irradiated sample with one pulse decreased. It increased gradually by increasing the number of pulses. Although the oxygen-based functional groups were observed in all laser-treated samples, their wettability differs significantly. The initial decrease in water contact angle could be related to production of polar functional groups on the polystyrene surface. More laser irradiation led to cleavage of chemical bonds and formation of a surface with regular ripples, air pockets, and hydrophobicity ([Mirzadeh and Dadsetan 2003]).

**Figure 5:**
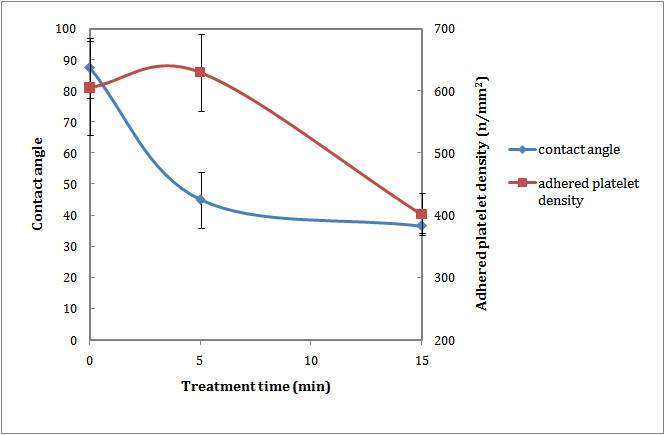
**Wettability and blood compatibility of PS after Ar plasma treatments.**

**Figure 6:**
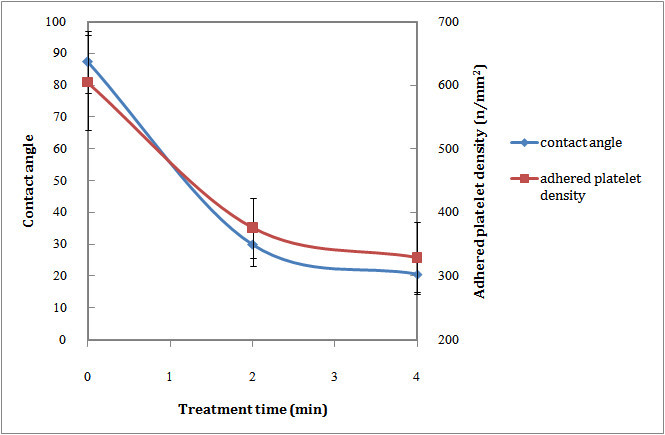
**Wettability and blood compatibility of PS after O**_**2**_ **plasma treatments.**

Figures 5 and 6 show that the water contact angle of both argon and oxygen plasma-modified samples decreased. It can be related to formation of polar functional groups on the polystyrene surface due to plasma treatment.

Comparison of the results obtained from the ATR-FTIR and wettability studies showed that in spite of almost similar chemical composition of the PS surfaces subjected to laser irradiation and Ar and O_2_ plasma treatments, their wettability is different significantly. It can be interpreted that the surface morphology of the laser- and plasma-treated samples had a great impact on polymer surface wettability.

### Blood compatibility studies

The LDH results for laser-irradiated samples are depicted in Figure 4. After laser irradiation with one pulse, the number of adhered platelets to the surface increased. By increasing the pulse numbers, the number of adhered platelets decreased significantly.

Study of the blood compatibility of Ar plasma surface-modified PS showed an initial increase in the number of adhered platelets to the surface and then decreased by increasing exposure time (Figure 5). Figure 6 depicts that after O_2_ plasma surface modification, the number of adhered platelets onto the surface decreased.

As the water contact angle cannot describe the wettability comprehensively, the surface tension of PS and water (*γ*_pw_) was used to explain the correlation between the wettability and blood compatibility:

1 $$\gamma_{\mathrm{pw}}=\gamma_{p}+\gamma_{w}-\sqrt[{2}]{\gamma_{p}^{d}\cdot \gamma_{w}^{d}}-\sqrt[{2}]{\gamma_{p}^{p}\cdot \gamma_{1w}^{p}}\text{.}$$

**Figure 7:**
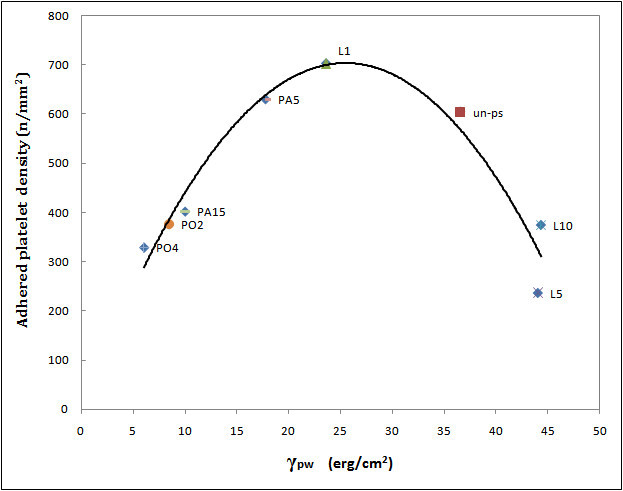
**Plot of the adhered platelet density as a function of** ***γ***_**pw**_.

In Equation 1, *γ*_pw_ is the surface tension between PS and water, *γ*_p_, *γ*_*p*_^*d*^, and *γ*_*p*_^*p*^ are the total, disperse part, and polar part of surface tension of PS, respectively. *γ*_w_, *γ*_*w*_^*d*^, and *γ*_*w*_^*p*^ represent the total, disperse part, and polar part of surface tension of water, respectively (Ikada et al. [1981]). The amount of *γ*_p_, *γ*_*p*_^*d*^, *γ*_*p*_^*p*^, and calculated *γ*_pw_ are presented in Table 1. The adhered platelet density (number of adhered platelets/mm^2^) is plotted against the calculated value of *γ*_pw_ (Figure 7).

**Table 1:** **The amount of** ***γ***_**p**_ **,** *γ*_*p*_^*d*^ **,** *γ*_*p*_^*p*^ **, and calculated** ***γ***_**pw**_ **of different samples**

Sample	***γ***_p_^d^ (erg/cm^2^)	***γ***_p_^p^ (erg/cm^2^)	***γ***_p_ (erg/cm^2^)	***γ***_pw_ (erg/cm^2^)
Un-PS	39.9	0.82	39.9	36.57
L1	49.7	5.04	49.7	23.63
L5	44	0.15	44	44.06
L10	36.8	0.07	36.8	44.36
PO2	49.6	16.1	49.6	8.48
PO4	57.7	22.3	57.7	6.08
PA5	45.9	7.6	45.9	17.78
PA15	51.2	14.6	51.2	10.03

*γ*_pw_, surface tension between PS and water; *γ*_p_, PS surface tension; *γ*_*p*_^*d*^, disperse part of PS surface tension; *γ*_p_^p^, polar part of PS surface tension.

As can be seen in Figure 7, the density of adhered platelets in the two extremes of hydrophilicity and hydrophobicity decreased. Ikada et al. (1983) proposed that when a superhydrophilic surface is subjected to the blood, a hydrous layer will be formed on the surface of the polymer and, as a consequence, platelets and proteins, which are initiators of the blood coagulation pathway, cannot come into contact with the surface. Thus, blood compatibility will increase. On the other hand, on surfaces with superhydrophobicity, protein adsorption on the surface, which is essential for platelet adhesion and activation, is low (Ikada et al. 1981).

Although samples L5 and L10 possess almost similar *γ*_pw_ of 43 erg/cm^2^, the adhered platelet density of L5 is less than that of L10. It can be due to the increase in the size of morphological patterns on the surface of sample L10. It has been shown that increasing the pulse number augmented the size of morphological patterns in a logarithmic manner. By increasing the valley width, the number of entrapped platelets in the roughness of the surface increased; thus, blood compatibility diminished (Dadsetan et al. 2001a).

Comparison of the encouraging results of blood compatibility and the biocompatibility results which were reported previously (Mirzadeh and Bagheri 2007) showed that by using these two techniques in different experimental conditions, we can achieve surfaces with different blood compatibility and biocompatibility characteristics. Thus, it is possible to use these two techniques to prepare polymeric surfaces for different applications.

## Conclusions

Laser treatment and RF plasma treatment were shown to induce a vast range of physiochemical properties onto the surface of PS and by regulating the treatment conditions; it is possible to achieve surfaces with hydrophobic and hydrophilic characteristics. The LDH results demonstrated that after laser irradiation and plasma treatment, the blood compatibility of the PS increased. Our results showed that the wettability of the PS surfaces strongly affects the blood compatibility of the treated PS surfaces. The best blood-compatible surfaces were L5 (laser irradiated with five pulses) and PO4 (oxygen plasma treated for 4 min) which had hydrophobic and hydrophilic surfaces, respectively.

## Methods

### RF plasma modification

In order to provide oxygen and argon plasma, a radio-frequency glow discharge plasma apparatus (EMITECH K1050X, Ashford, UK) was applied. Samples, after washed with deionized water and dried, were exposed to oxygen and argon plasma individually. The treatment condition for each sample is presented in Table 2.

**Table 2:** **Experimental conditions of different samples**

Sample codes	Method of treatment	Gas of chamber	Treatment time/pulse number
Un-PS	Untreated	-	-
L1	Laser	ArF	1 pulse
L5	Laser	ArF	5 pulse
L10	Laser	ArF	10 pulse
PO2	RF plasma	O_2_	2 min
PO4	RF plasma	O_2_	4 min
PA5	RF plasma	Ar	5 min
PA15	RF plasma	Ar	15 min

### Laser irradiation

ArF excimer laser (LAMBDA PHYSIK LPX® 210, Fort Lauderdale, FL, USA) with *λ* of 193 nm, laser fluence of 190 mJ/cm^2^, and repetition rate of 1 Hz was applied to treat the surface of polystyrene films. Both sides of the samples were modified. All modified samples are presented in Table 2.

### Surface characterization

An atomic force microscope (AFM; Park Scientific Instruments Autoprobe, Sunnyvale, CA, USA) was used to investigate morphological changes of the polystyrene surface after laser and plasma modification. Sessile drop method with two solvents (water and diiodomethane) was applied to measure static contact angles using Kruss G10 equipment (Hamburg, Germany). All results are average of five measurements. The Owens and Wendt method (Owens and Wendt 1969) was used to calculate the polar part (*γ*^p^), disperse part (*γ*^d^), and surface tension (*γ*) of the samples.

### Blood compatibility tests (LDH method)

Platelet-rich plasma (PRP) was obtained from healthy human venous blood. A Coulter counter (type 4) was used to count the platelet numbers of PRP. Then, it was adjusted to 300,000 platelets/mm^3^. Each sample with an area of 1 cm^2^ was subjected to 1 mL of PRP for 1 h at 37°C. After being washed with phosphate-buffered saline (PBS), 2 mL of lysis buffer (0.5% Triton X-100) in PBS was added onto the films in a test tube to determine the number of adhered platelets. The lysis was allowed to proceed for 1 h at room temperature to ensure complete platelet disruption. The lactate dehydrogenase (LDH), as reagent activity of lysate, was measured by addition of 0.3 mL of substrate buffer to the tube. The change in ultraviolet absorption at 340 nm was measured immediately, using an ultraviolet spectrophotometer. LDH calibration curve was obtained by measuring the enzymatic activity of a set of samples with a known concentration of platelets in PBS buffer under the same condition as the tested films (Dadsetan et al. 2001aKhorasani and Mirzadeh2004a).

## Authors' information

HM is a professor at the Departments of Polymer Engineering and Biomedical Engineering at Amirkabir University of Technology. He has published more than 140 peer-refereed journal articles in the fields of polymeric biomaterials and tissue engineering.

## Authors’ original submitted files for images

Below are the links to the authors’ original submitted files for images. Authors’ original file for figure 1 Authors’ original file for figure 2 Authors’ original file for figure 3 Authors’ original file for figure 4 Authors’ original file for figure 5 Authors’ original file for figure 6 Authors’ original file for figure 7 Authors’ original file for figure 8 Authors’ original file for figure 9
