# Supplementary figures and images for: Polystyrene surface modification using excimer laser and radio-frequency plasma: blood compatibility evaluations
Source: Prog Biomater. 2012 Nov 14;1:4. doi: 10.1186/2194-0517-1-4 (PMC5120664; doi:10.1186/2194-0517-1-4)

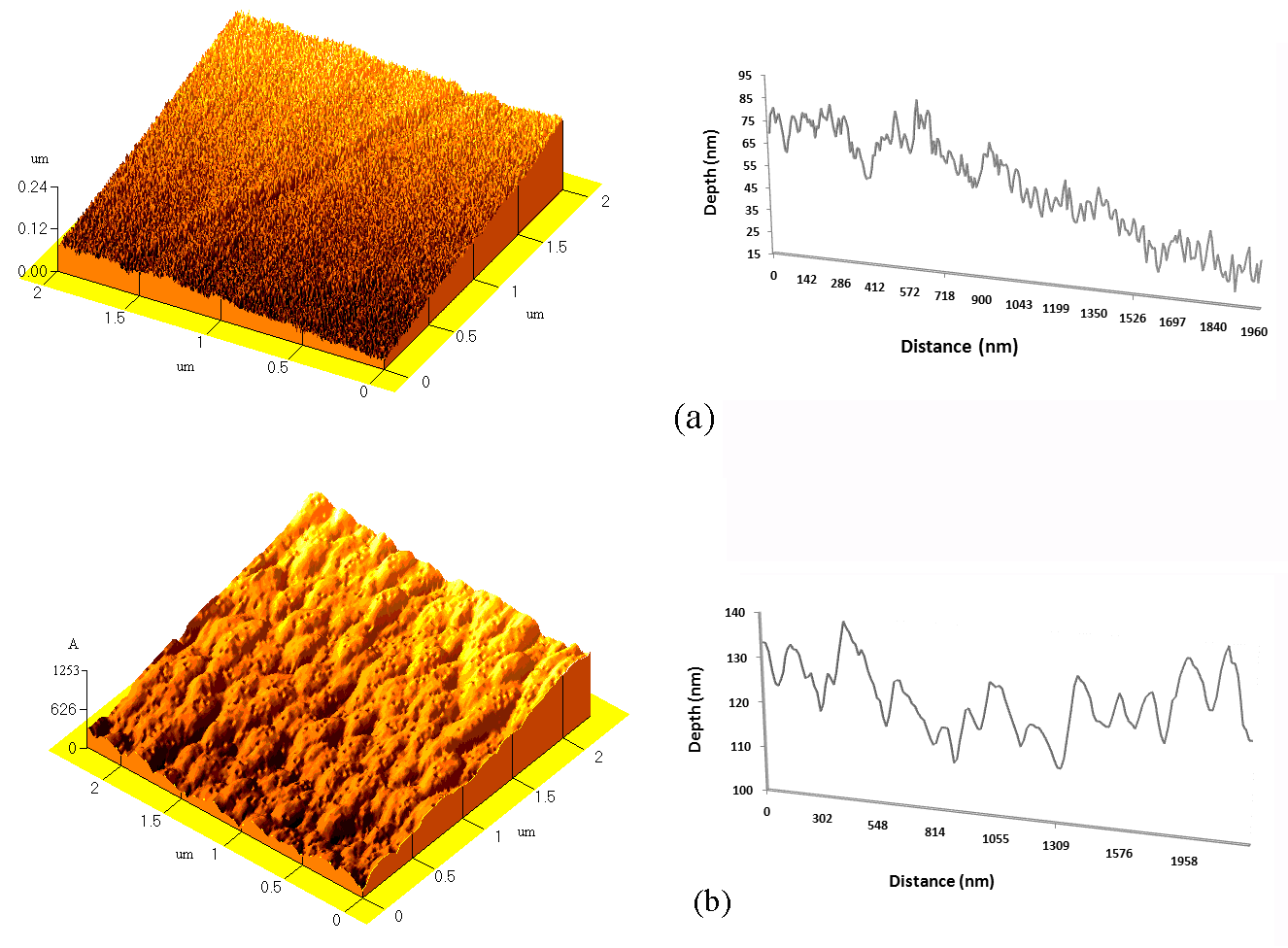

Supplement: Supplementary file 1 — Authors’ original file for figure 1 [file 40204_2012_4_MOESM1_ESM.tiff]

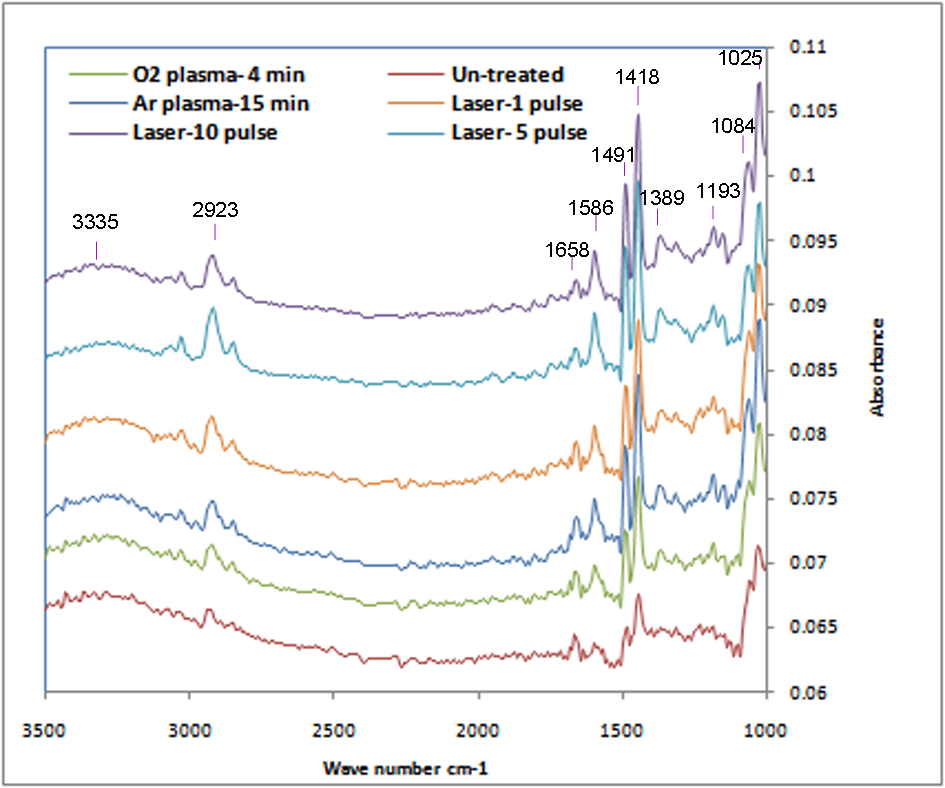

Supplement: Supplementary file 3 — Authors’ original file for figure 3 [file 40204_2012_4_MOESM3_ESM.tiff]

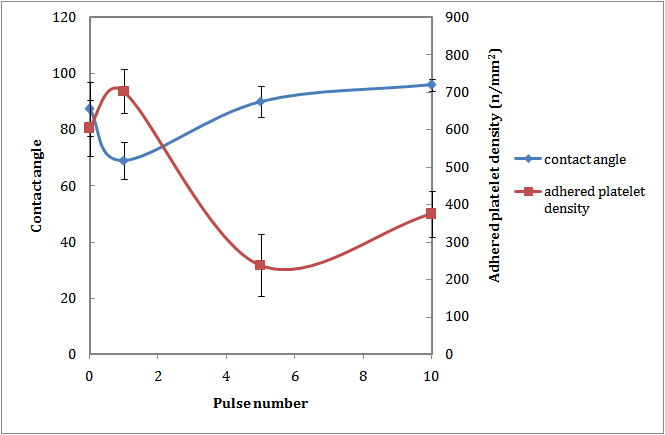

Supplement: Supplementary file 4 — Authors’ original file for figure 4 [file 40204_2012_4_MOESM4_ESM.tiff]

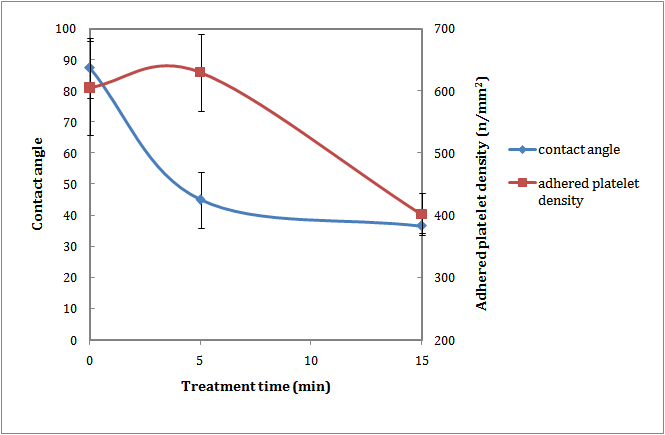

Supplement: Supplementary file 5 — Authors’ original file for figure 5 [file 40204_2012_4_MOESM5_ESM.tiff]

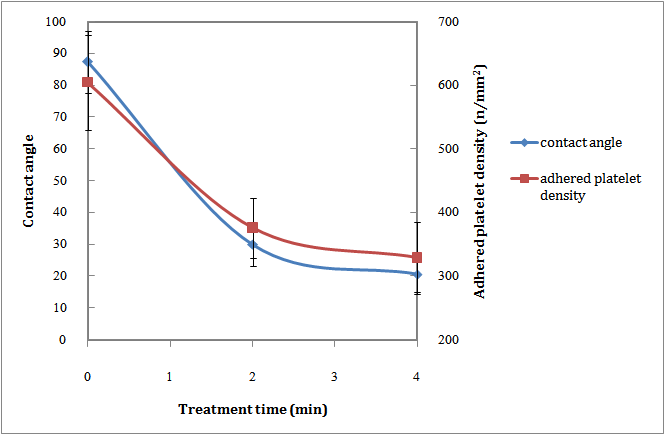

Supplement: Supplementary file 6 — Authors’ original file for figure 6 [file 40204_2012_4_MOESM6_ESM.tiff]

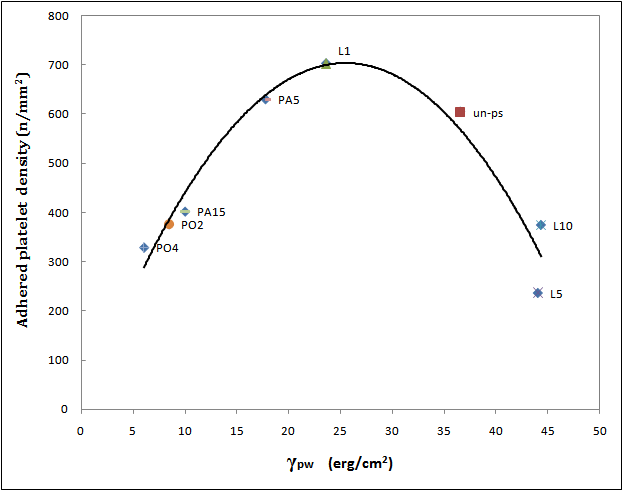

Supplement: Supplementary file 7 — Authors’ original file for figure 7 [file 40204_2012_4_MOESM7_ESM.tiff]
